# Public perception of drinking water safety in South Africa 2002–2009: a repeated cross-sectional study

**DOI:** 10.1186/1471-2458-12-556

**Published:** 2012-07-27

**Authors:** Jim A Wright, Hong Yang, Ulrike Rivett, Stephen W Gundry

**Affiliations:** 1 Geography and Environment, University of Southampton, University Road, Southampton, SO17 1BJ, UK; 2 iCOMMS, Department of Civil Engineering, University of Cape Town, Rondebosch, 7700, Cape Town, South Africa; 3 Water and Health Research Centre, Merchant Venturers Building, University of Bristol, Bristol, BS8 1UB, UK

**Keywords:** Household survey, Water safety, Drinking water, Water quality, Consumer perception, South Africa, Cholera

## Abstract

**Background:**

In low and middle income countries, public perceptions of drinking water safety are relevant to promotion of household water treatment and to household choices over drinking water sources. However, most studies of this topic have been cross-sectional and not considered temporal variation in drinking water safety perceptions. The objective of this study is to explore trends in perceived drinking water safety in South Africa and its association with disease outbreaks, water supply and household characteristics.

**Methods:**

This repeated cross-sectional study draws on General Household Surveys from 2002–2009, a series of annual nationally representative surveys of South African households, which include a question about perceived drinking water safety. Trends in responses to this question were examined from 2002–2009 in relation to reported cholera cases. The relationship between perceived drinking water safety and organoleptic qualities of drinking water, supply characteristics, and socio-economic and demographic household characteristics was explored in 2002 and 2008 using hierarchical stepwise logistic regression.

**Results:**

The results suggest that perceived drinking water safety has remained relatively stable over time in South Africa, once the expansion of improved supplies is controlled for. A large cholera outbreak in 2000–02 had no apparent effect on public perception of drinking water safety in 2002. Perceived drinking water safety is primarily related to water taste, odour, and clarity rather than socio-economic or demographic characteristics.

**Conclusion:**

This suggests that household perceptions of drinking water safety in South Africa follow similar patterns to those observed in studies in developed countries. The stability over time in public perception of drinking water safety is particularly surprising, given the large cholera outbreak that took place at the start of this period.

## Background

The general public have long been recognised as an important stakeholder in the management of drinking water supplies [1]. Public acceptability of drinking water forms part of the *World Health Organization*’s *Guidelines for Drinking Water Qualit*y [2], which incorporates guidelines on consumer acceptability of taste, colour and odour. In developed countries, studies of drinking water safety perception have been particularly focused around bottled water consumption [3,4], private supplies [5] and trust in municipal water [6], desalinated water [7] and recycled water [7,8]. In developing countries, such studies have examined water safety perceptions in relation to home water treatment uptake [9,10], such as household filtration or chlorination. A study of Sri Lankan households, for example, found that a higher perceived contamination risk increased the probability of households boiling or filtering drinking water [11]. Where households have a choice of several different water sources (e.g. boreholes versus vended water), perceived drinking water safety may influence source choice [12] and thereby the financial sustainability of water services through tariff recovery.

More recently, information has been recognised as an intervention that can shape public perceptions of drinking water safety, both in South Africa and elsewhere. Where water quality is poor, the release of water quality information to consumers may help to promote home water treatment [13] or use of safer source types, and potentially promote public pressure on suppliers to improve service quality. Where water quality is acceptable, releasing water quality information may potentially help reassure consumers and improve customer relations. Experimental evidence suggests that information provision to consumers may alter their perception of recycled drinking water safety [14], although there is weaker evidence for households switching sources after being provided with information about arsenic or microbiological water contamination [15].

In South Africa, government initiated a Blue Drop scheme in 2009 [16], which uses media releases to inform the public about the water quality-related performance of Water Service Providers and Authorities. Such releases are made through various media channels (e.g. [17]). Rand Water, a contracted water service provider, also has a public outreach programme that involves schools outreach, a web site (http://www.reservoir.co.za), call centre, and a volunteer household water testing programme. In the latter Tap Analysis Programme, householders can volunteer to test their own tapwater using easy-to-use kits and then receive individualised results once samples have been processed by an appropriate laboratory [18].

In both developed and developing countries, most studies of public drinking water safety perceptions have been cross-sectional in nature [19,20] and have often focused on small groups of consumers [21,22]. This raises the question as to whether public perceptions of drinking water safety remain constant over time or whether they fluctuate in response to health promotion campaigns, initiatives like the Blue Drop scheme, or events such as water-borne disease outbreaks. In this study, our objective is to assess the trends in the public perception of drinking water safety over time, by drawing on a series of nationally representative studies conducted in a middle income country, South Africa.

## Methods

### Data sources

The General Household Survey (GHS) is a nationally representative survey that has taken place every July in South Africa since 2002. The survey acts as a replacement for the earlier October Household Survey, which ran from 1993 to 1999 [9]. Data can be downloaded for research purposes from the South African Data Archive (http://www.nrf.ac.za/sada/). The content of the survey is decided by an inter-ministerial panel and consists of a core set of questions administered every year, together with themed questions that vary from one year to the next. In each year from 2002 until 2009, with the exception of 2004, there have been questions relating to drinking water safety, clarity, odour, and taste. In particular, the survey included the question ‘Is the water from the main source of drinking water safe to drink?’, which was not included in the earlier October Household Survey. In most years, there have also been questions relating to drinking water treatment. The survey typically comprises around 25,000 households each year and rather than following the same households over time, a new set of households is recruited each year of the survey.

The survey is designed to provide representative estimates of household characteristics at national and provincial level, but not for smaller geographic units. The geography of the survey has varied in line with administrative reforms within South Africa. For 2002 and 2003, magisterial districts (of which there were 367 nationally) are the smallest geographical units to which data can be related. 331 of these magisterial districts were sampled in the 2003 GHS. In later years (2005–2007), survey households can be related to the newer administrative geography of South Africa (4 Metropolitan and 48 District municipalities).

### Statistical analysis

#### Trends in perceived drinking water safety

We examined trends in the overall proportion of the South African population who felt their water was safe between 2002 and 2009. Since the proportion of households using unsafe sources such as streams / rivers in the GHS has fallen over time [23] in line with South African’s water service delivery programme, we also examined these trends separately for the types of water source in most widespread use.

Since South Africa is known to have experienced at least one cholera outbreak immediately before the study period, which took place in 2000–02 and particularly affected KwaZulu-Natal [24], we also obtained annual statistics on cholera cases reported by South Africa from the World Health Organization’s *Weekly Epidemiological Record*[25,26].

#### Modelling perception of drinking water safety

We examined the following possible groups of water service characteristics that could account for households perceiving their drinking water as unsafe (in descending order of likely importance, following [1]):

*Water taste, colour and odour*: Previous studies of bottled water use by consumers in developed countries have found such organoleptic water characteristics to be important [27], whilst in India, rural respondents cited clarity, lack of odours, and an unsalty taste as being characteristics of safe water [21].

*Water supply type*: Water supply type provides contextual grounds for households to assess drinking water safety, as evidenced by respondents reported in Banda et al. (2007) who considered boreholes to be associated with safe water. We therefore distinguished piped supplies from boreholes, rainwater and tanked water, and surface waters, springs and wells by grouping together the relevant GHS source types. Since perceived control of supply systems has sometimes also been associated with water safety [28], we further distinguished public / communal taps and neighbours’ taps from piped water on a household’s premises.

*Water interruptions*: These provide a further contextual indicator of drinking water safety and there is some evidence from South Africa that dissatisfaction with supply interruptions could extend to other aspects of service delivery such as water quality [29].

We also examined several groups of household characteristics that could be associated with perceived drinking water safety (again in descending order of importance):

*Information deprivation*: Since there is some evidence that complaints to South African water providers increase in response to media coverage of the water sector [18], we examined information access in shaping perceptions of drinking water safety. Gordon et al. [30] have developed a composite measure of information deprivation, which is based on access to newspapers, radio, television, and telephone, and we used a similar metric here, counting the number of such information sources a household had access to.

*Residence in a cholera-affected province*: The South African government initiated public health campaigns to promote home water treatment in cholera-affected areas [31], which may have raised awareness of unsafe drinking water. We therefore examined residence in a cholera-affected province as a possible influence on perceived drinking water safety. Over 97% of the 17,902 cholera cases reported between August 2001 and July 2002 (the date of the GHS) were in KwaZulu-Natal and Eastern Cape Provinces [32], so we generated a dummy variable to represent these provinces. Although a cholera outbreak also took place in 2008 centred on Limpopo and Mpumulanga Provinces, cases were only reported in November and December, after the GHS took place in July [33]. We therefore did not use a provincial dummy variable in this year.

*Household expenditure*: As a measure of socio-economic status, we focused on household expenditure rather than income, since such measures are much more reliable and are easier to collect than income, especially in most rural settings [34].

*Ethnicity, educational level, and gender of household head*: Ethnicity was considered fundamental by Maharaj and Pietersen [35] when consulting South Africa’s population over water sector policy, whilst Anderson et al. [9] found attitudes to water pollution varied between African and other ethnic groups in South Africa. Pradhan [36] describes the proportion of households linking contaminated water with disease as varying by educational level and gender, independent of ethnicity.

We hypothesised that socio-economic and demographic characteristics would be weakly associated with drinking water safety perceptions, following evidence from developed countries [1].

The relationship between perceived drinking water safety and the set of explanatory variables was modelled using logistic regression (in Stata version 11: *svy: logit*) for two survey years, namely 2002 and 2008. These years were chosen as being far apart, yet having sufficiently similar questionnaire content to enable a comparable analysis to be undertaken. For both years, backwards hierarchical stepwise regression [37] was used to examine the contribution of each group of covariates above, beginning with those of hypothesised lowest importance. Each group’s significance was examined via a *Wald* test and discarded if non-significant at the 99% level. Individual covariates within retained groups were subsequently tested in the same way.

Pooling data from all survey years proved problematic because of changes in sample weight construction after 2007 and minor changes in the wording of smell and water clarity questions after 2004. Nonetheless, we were able to pool the survey data into three periods for 2002–3, 2005–7, and 2008–9. To test for inter-year differences in perceived drinking water safety, we fitted a logistic regression that included source type, organoleptics, and yearly dummy variables as covariates to these pooled data sets.

## Result

### Trends in public perceptions of drinking water safety

**Figure 1:**
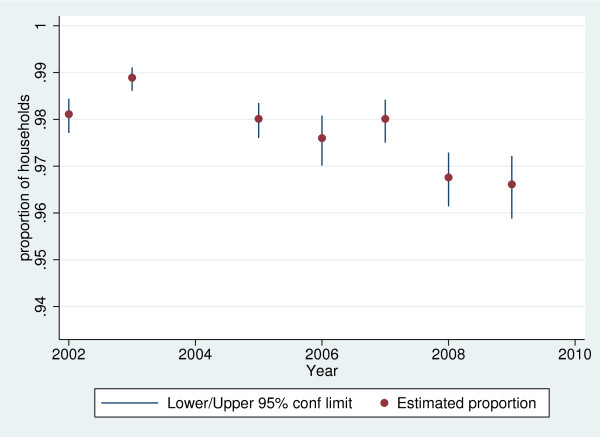
**Short title: Proportion of households with water piped to dwelling who believe water is safe, 2002–2009.** Detailed legend: Proportion of South African households with water piped to dwelling who believe their drinking water is safe to drink, 2002–2009 (based on 10,281 households in 2002, 10,411 in 2003, 9,721 in 2005, 10,073 in 2006, 10,541 in 2007, 9,342 in 2008 and 9,651 in 2009. Note: there was no question about drinking water safety in 2004).

**Figure 2:**
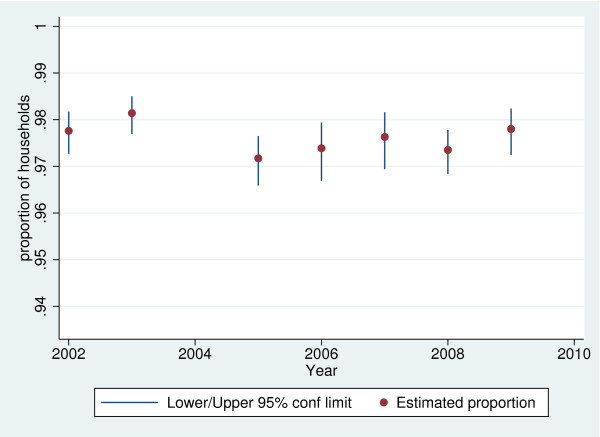
**Short title: Proportion of households with water piped to yard who believe water is safe, 2002–2009.** Detailed legend: Proportion of South African households with water piped to their yard or onsite who believe their drinking water is safe to drink, 2002–2009 (based on 7,825 households in 2002, 8,029 in 2003, 8397 in 2005, 8,269 in 2006, 8,764 in 2007, 7,228 in 2008 and 7,740 in 2009).

**Figure 3:**
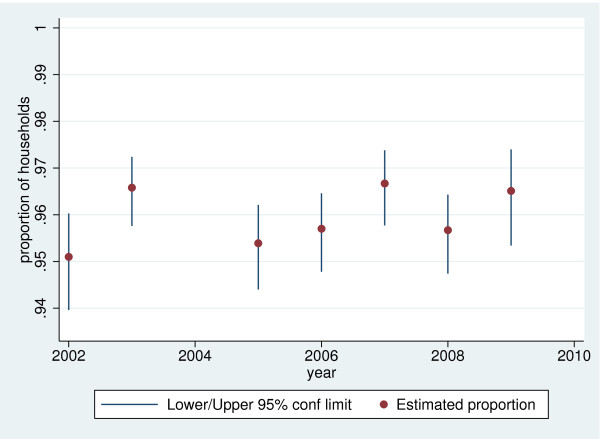
**Short title: Proportion of households using public or communal taps who believe water is safe, 2002–2009.** Detailed legend: Proportion of South African households using public or communal taps who believe their drinking water is safe to drink, 2002–2009 (based on 3,392 households in 2002, 3,720 in 2003, 4,652 in 2005, 4,520 in 2006, 4,915 in 2007, 4,004 in 2008 and 4,313 in 2009).

**Figure 4:**
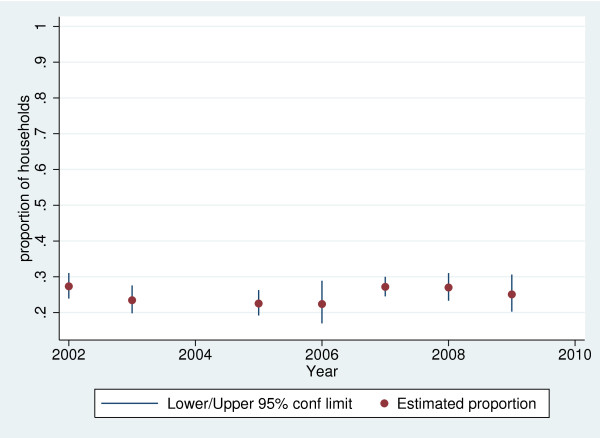
**Short title: Proportion of households using flowing surface water who believe drinking water is safe, 2002–2009.** Detailed legend: Proportion of South African households using flowing surface waters from streams and rivers who believe their drinking water is safe to drink, 2002–2009 (based on 1,422 households in 2002, 1, 191 in 2003, 1,564 in 2005, 1,407 in 2006, 1,475 in 2007, 975 in 2008 and 1,107 in 2009).

The proportion of households who believed their drinking water was safe rose from 89.6% (95% confidence limits 88.9 - 90.3%) in 2002 to 92.7% (95% confidence limits 92.0 - 93.4%) in 2009. An analysis of year-on-year variation in household beliefs about drinking water safety is shown in Figures 1 to 4, for the four most common types of supply in South Africa. The confidence limits reflect the sampling error associated with the complex survey design. It is difficult to discern a trend in the perceived safety of flowing surface water from streams or rivers, public or communal taps, and water piped into the yard or onsite. There was an increase in the (relatively small) proportion of those using in-house, piped supplies who believed their water was unsafe.

**Figure 5:**
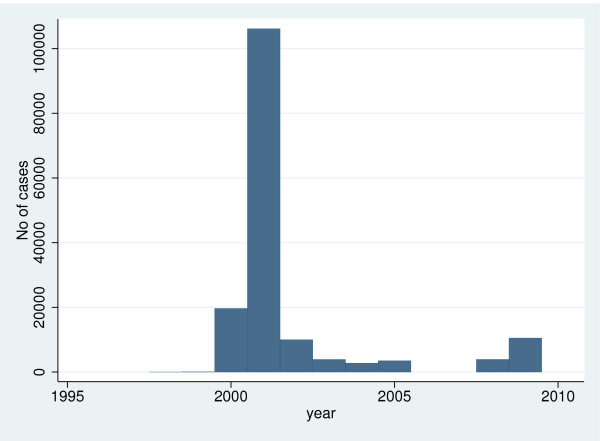
**Short title: Reported South African cholera cases, 1996–2010.** Detailed legend: Number of South African cholera cases reported to the World Health Organization, 1996–2010 (data sources [25,26]).

Figure 5 shows the number of cholera cases reported to the WHO from 1996 to 2010. In several years, namely 1996–97, 2006–07, and 2010, South Africa did not report any cholera cases. The annual number of cholera cases reported to WHO peaked in 2001, with large numbers of cases also reported in 2000, 2002, and 2009. The 2001 outbreak was particularly concentrated in KwaZulu-Natal province (Mendelsohn and Dawson, 2008). Despite the very large spike in the number of cholera cases in 2001 in particular, there was no noticeable corresponding spike in the number of South Africans who believed their water was unsafe to drink in 2002, either in the country as a whole or in KwaZulu-Natal specifically.

### Factors influencing perceived drinking water safety

In order to further understand possible changes in perceived drinking water analysis over time, we conducted a more in-depth analysis of 2002 and 2008. Table 1 summarises the characteristics of households sampled through the GHS in both of these years. There was a slight reduction in the proportion of households reporting water that was unclear and with poor taste or odour. The proportion of households drinking from unimproved sources such as streams and dams also decreased. Against this, the proportion of households experiencing interruptions to their water supply increased from 2002 to 2008.

**Table 1:** Summary of household and water supply characteristics from the 2002 and 2008 general household surveys

	**Percentage of households (observations unweighted to adjust for sample design)**	
	**2002**	**2008**
No. households	26,243	24,222
Variable		
Water supply characteristics		
Water free from odours	88.85	91.08
Water clear	90.52	91.29
Water tastes good	89.22	90.72
Private piped supply	67.56	70.46
Public or communal taps / neighbour’s piped supply	16.19	18.09
Borehole	4.41	3.14
Rainwater or water carrier	1.24	1.54
Other sources (e.g. river, stream, dam, well, spring)	10.54	6.43
Supply interruptions		
Daily	2.50	3.79
Weekly	5.45	8.08
Monthly	8.72	11.22
6-monthly	8.62	8.35
Yearly	3.80	4.17
Almost never	54.03	37.87
Household characteristics		
Household head male	62.18	60.90
Household head ethnic group:		
Black African	77.12	76.47
Coloured	7.91	8.29
Indian or Asian	2.40	2.63
White	12.57	12.61
Household head educational level		
No education	16.16	14.17
Attended primary education	28.70	24.43
Attended secondary education or higher	53.73	55.12
Household monthly expenditure (South African Rand)		
< 400	31.29	9.38
400 – 799	27.17	23.13
800 – 1199	11.94	19.12
1200 – 1800	7.08	12.42
1800 – 2500	5.54	8.57
2500 – 5000	7.17	11.44
5000 – 10000	4.69	7.99
>10000	1.66	5.33

The relationship between these household characteristics and perceived drinking water safety were then examined using hierarchical stepwise logistic regression, as noted earlier. This process involved an assessment of the impact of successively removing groups of covariates from the model. For 2008, all groups of covariates were entered in the first step of the regression analysis with household head characteristics being discarded as insignificant (F = 0.71; p = 0.64) and expenditure discarded in the second step (F = 1.78; p = 0.13). At the third step, the information deprivation index was discarded (F = 2.34; p = 0.13) and at the fourth step, supply interruption variables were discarded (F = 1.93; p = 0.15). The group of variables relating to water source type were retained in the fifth step (F = 103.7; p < 0.001), as were the organoleptic variables in the sixth step (F = 628.7; p < 0.001). For 2002, household head characteristics were retained at the first step (F = 2.95, p = 0.007), but household expenditure, the information deprivation index, supply interruptions and cholera-affected provinces were discarded in in the subsequent four steps (F = 1.57, 2.01, 0.62, and 5.65; p = 0.14, 0.16, 0.68 and 0.02, respectively). In the final two steps, source types and organoleptic properties were retained (F = 140.1 and 586.8 respectively; p < 0.001). With models for both years, Wald test statistics for individual variables within these groups were then examined, as were possible interactions between them. Odds ratios for coefficients included in the final logistic regression models resulting from this process are shown in Table 2. In broad terms, the odds ratios for household and water supply characteristics were consistent between 2002 and 2008, with water clarity and good taste being particularly strongly associated with perceived drinking water safety, as evidenced by the high odds ratios for these characteristics. This association between taste, clarity and safety was weaker for water drawn from wells, springs, dams and rivers. When a regional dummy variable was added to the 2002 model to represent KwaZulu-Natal province, the centre of the cholera outbreak, this was not significantly associated with perceived drinking water safety.

**Table 2:** Odds ratios from a logistic hierarchical stepwise regression model, predicting perceived drinking water safety from water supply and household characteristics (based on 26,076 households in the 2002 and 24,056 households in the 2008 general household survey)

**Characteristic**	**2008**			**2002**		
	**Odds ratio**	**T statistic**	**P value**	**Odds ratio**	**T statistic**	**P value**
Water clear	10.44	11.49	0	22.60	13.4	0
Water tastes good	14.08	15.58	0	8.24	9.19	0
Water free from odours	2.47	5.15	0	4.07	6.15	0
Borehole	0.35	−3.48	0.001	0.20	−6.07	0
Rainwater or tanker water	0.24	−4.44	0	0.13	−6.3	0
Other non-piped water sources (i.e. wells, rivers, dams, springs, stream, etc.)	0.25	−6.5	0	0.10	−9.23	0
Water clear and free from odours	3.85	5.39	0	2.22	3.01	0.003
Clear water from other non-piped sources	0.23	−5.07	0	0.44	−3.1	0.002
Water from other non-piped sources tastes good	0.42	−3.42	0.001	0.43	−2.9	0.004
Water from other non-piped sources free from odours				0.34	−3.92	0
Head of household ethnicity - coloured				3.70	3.39	0.001
Public or neighbour’s tap				0.52	−2.92	0.004

To illustrate the nature of the fitted model more clearly, Table 3 shows the probability of a household perceiving its drinking water supply as unsafe, as predicted by the model for 2008. The table shows only combinations of water source type and water characteristics that were experienced by more than 100 households. The importance of water taste, odour and clarity is apparent from this table. For example, the model suggests that only 14.3% of households believe unclear piped water with noticeable odours and poor taste is safe, yet this figure rises to 99.6% if piped water is clear, tastes good and is odour free. Table 3 shows that the effect is mediated by source type: water that tastes good, is free from odours and is clear has a 99.6% probability of being perceived as safe if it is from a piped supply. If such water comes from surface waters, wells or springs, the probability falls to 84.6%.

**Table 3:** Relationship between perceived drinking water safety and water supply characteristics, 2008 (as predicted by the final logistic regression model)

**Free from odour**	**Good taste**	**Clear**	**Supply type**	**% Households perceiving water safe**	**No. households**
N	N	N	Other (i.e. surface waters, Wells, and springs)	3.9	727
Y	N	N	Other (i.e. surface waters, Wells, and springs)	9.0	122
N	N	N	Piped supplies	14.3	479
Y	N	N	Piped supplies	29.2	174
N	N	Y	Piped supplies	63.5	138
Y	Y	Y	Other (i.e. surface waters, Wells, and springs)	84.6	406
Y	Y	N	Piped supplies	85.3	309
Y	N	Y	Piped supplies	94.3	333
N	Y	Y	Piped supplies	96.1	310
Y	Y	Y	rain / tanker water	98.3	280
Y	Y	Y	Boreholes	98.8	628
Y	Y	Y	Piped supplies	99.6	19413

When a logistic regression model was fitted to pooled data for 2002–3 combined, 2005–7 combined, and 2008–9 combined, there was no evidence for yearly differences in perceived drinking water safety within these periods (T = 1.33, p = 0.18 for the dummy variable representing 2009; T = −0.41 p = 0.68 for 2007, T = −0.67; p = 0.51 for 2006; T = 0.66 p = 0.51 for 2003).

## Discussion

Although there have been a number of large-scale studies of public perceptions of drinking water safety in developed countries, this issue has been less widely studied in low and middle income countries, often through smaller scale studies [1]. This study focused on a large-scale multi-year data set for South Africa, a middle income country but one with high levels of inequality. It confirms the finding from high income countries that consumers predominantly respond to organoleptic signs of contamination in their perception of drinking water safety, with socio-economic and demographic characteristics being comparatively less important. This is consistent with findings in other middle income countries, notably an urban Ukrainian population, which rated drinking water safety as fair to unsafe and also rated organoleptic properties as fair to poor [20].

Of the socio-economic and demographic characteristics considered, only ethnicity was included in the final model from the 2002 General Household Survey (Table 2). Despite the high levels of inequality, there was no evidence here that perception of drinking water safety differed by socio-economic status as measured through household expenditure. Of the three organoleptic properties, clarity and taste appeared more important than odour (Tables 2 and 3). However, despite this strong relationship, the link between organoleptic properties and perceived drinking water safety is likely to be complex. In a survey of tap water consumers in Cape Town, for example, nine out of 21 respondents complaining of poor taste cited too much chlorine as the cause [38]. Similarly, five out of nine respondents (in formal residential areas) detecting an odour attributed this to chlorine.

There was some evidence that contextual clues – most notably the source type – were linked to perceived drinking water safety. There was also some evidence for a link between perceived control over water supplies and their perceived safety. In particular, in 2002, tapwater users relying on a public or neighbour’s tap were less likely to consider their water safe.

There has similarly been relatively little work exploring the change in consumer perceptions of drinking water safety over time. After controlling for the expansion in improved water supply access over the study period through the pooled survey data analysis, household perceptions of drinking water safety appear stable over time, although small changes in perceptions would be difficult to detect because of survey sampling errors. There was however an increase in the small number of consumers with water piped into the dwelling, who considered their water unsafe between 2003 and 2009 (Figure 1). A slight decline in such piped water that was free from organoleptic signs of contamination over this period is likely to account for the apparent trend in this group. This finding is perhaps unexpected, given the widespread cholera outbreak that affected South Africa in 2000–02 (Figure 5) and the link between prior history of water-borne disease and perceived drinking water safety [1]. Elsewhere, a seasonal increase in household water treatment has been noted in Madagascar in response to a perceived increase in cholera risk [39], but there was no such discernible link here. This was despite a ministry of health campaign using radio, television and pamphlets warning of unsafe sources [40].

Other recent studies have used the GHS to examine other aspects of drinking and water resource management. The 2004 GHS has previously been used to explore attitudes towards environmental water pollution and uptake of home water treatment [9]. Similarly, the 2002 GHS has been used to assess characteristics of households lacking access to safe water [41] as well as patterns of water supply cut-offs [29]. Given that home water treatment involves households taking action to reduce consumption of unsafe water, this may provide additional insights into perceived drinking water safety.

In theory, it would be possible to compare the perceived safety of different source types with scientific measures of water quality from monitoring systems and other surveys of water supplies. Examples of such data include that published under the Blue Drop scheme [16] and earlier reports such as MacIntosh and Colvin [42] for groundwater or Ehlers et al. [43] for bottled water. However, such comparisons are likely to be problematic and were not attempted here because:

Many potential sources of water quality data are not restricted to the point-of-use, but also entail sampling of untreated source water or water within distribution systems.

Professional assessments of drinking water safety often rely on both testing of multiple water quality parameters and sanitary risk inspection. However, many data sources only include a subset of these parameters and may omit risk inspections altogether. Not only is there a problem of partial coverage of drinking water safety parameters in some data sets, but finding an appropriate way of integrating these measurements into a single composite metric of water safety (for example, by choosing the lowest water safety band across all measured parameters) is also potentially difficult.

Whilst there are monitoring systems and sample surveys available, few are designed to provide nationally or provincially representative estimates.

Since consumer perception of drinking water safety seems largely dependent on the organoleptic properties of water supplies, this implies that information about water quality disseminated through the media (such as through the South African Blue Drop reports) may not alter public opinion on safe water greatly. However, although the perceptions of the general public may be relatively intransigent, such campaigns may still be successful in mobilising specific community activists, consumer groups and in motivating water sector professionals to improve on service levels.

Because the study relied on a secondary data set, a number of potentially important influences on perceived drinking water safety were omitted from this analysis. Such influences include trust in water providers like municipalities; information exchange with neighbours and other contacts; previous experience of water borne diseases such as cholera; recent changes in the nature of drinking water provision; and household perceptions of risk [1]. Unlike some other studies [27], we did not attempt to model the multiple inter-dependencies between potential influences on perceived drinking water safety, such as taste, clarity and source type. The potential for response bias [44] arising from the interaction between interviewer and interviewee in such government-sponsored surveys is also well documented, though the direction of any bias is unclear in the GHS. Although the surveys analysed covered many households, because of their inherent design, we were limited in our ability to identify specific locations where confidence in supplies was low, disaggregate results for specific source types, or detect slight changes in perceived water safety over time. More generally, the survey is useful for understanding general changes in public confidence in water supplies over time, but less useful in understanding the perceptions of specific sub-groups of the population who may be particularly important in holding service providers to account.

It is unclear how far South African media coverage of the water sector may have varied over the period of study or whether the relatively stable consumer perception of drinking water safety mirrors media coverage over the period. However, it seems likely that the cholera outbreak of 2000–02 in particular would have attracted substantial [45] media attention. However, media reports related to the water sector and outbreaks of diseases such as cholera require further investigation. There may also be scope in future research to examine specific sub-groups of the population such as those complaining directly to utilities [18] or awareness of specific campaigns to disseminate water quality information, such as the Blue Drop initiative.

## Conclusion

This study suggests public perceptions of drinking water safety in South Africa have remained remarkably consistent over the period 2002–09. An apparent increase in perceived drinking water safety was accounted for by greater improved supply coverage and associated improvements in water taste, odour and clarity. The consistency over time in perceived drinking water safety is particularly remarkable, given a large cholera outbreak in 2000–2002. Water clarity, odour, and taste were strongly associated with perceived drinking water safety in both 2002 and 2008. As with many studies conducted in high income countries, few socio-economic household characteristics were associated with perceived drinking water safety.

Recently, the South Africa government has publicly released water quality management information, hoping to trigger greater consumer pressure on suppliers to improve services. Given that historically public perception of drinking water safety has remained stable, it remains to be seen whether such information dissemination will result in greater pressure from either the public or consumer groups for better water services.

## Competing interests

The authors declare that they have no competing interests.

## Authors’ contributions

JAW and SWG conceived the study and participated in its design. JAW coordinated the study throughout. JAW and UR collated and analysed the cholera data for South Africa. JAW and HY generated statistical code and conducted the survey data analysis. All authors interpreted the data. JAW and HY wrote the first draft and all authors critically revised the draft and approved the final manuscript.

## Pre-publication history

The pre-publication history for this paper can be accessed here:

http://www.biomedcentral.com/1471-2458/12/556/prepub
